# Supplementary material for: Treatment of COVID-19 during the Acute Phase in Hospitalized Patients Decreases Post-Acute Sequelae of COVID-19
Source: J Clin Med. 2023 Jun 20;12(12):4158. doi: 10.3390/jcm12124158 (PMC10299438; doi:10.3390/jcm12124158)
Supplement: Supplementary file 1 [file jcm-12-04158-s001.zip › jcm-2387544-supplementary.pdf]

Supplementary Materials

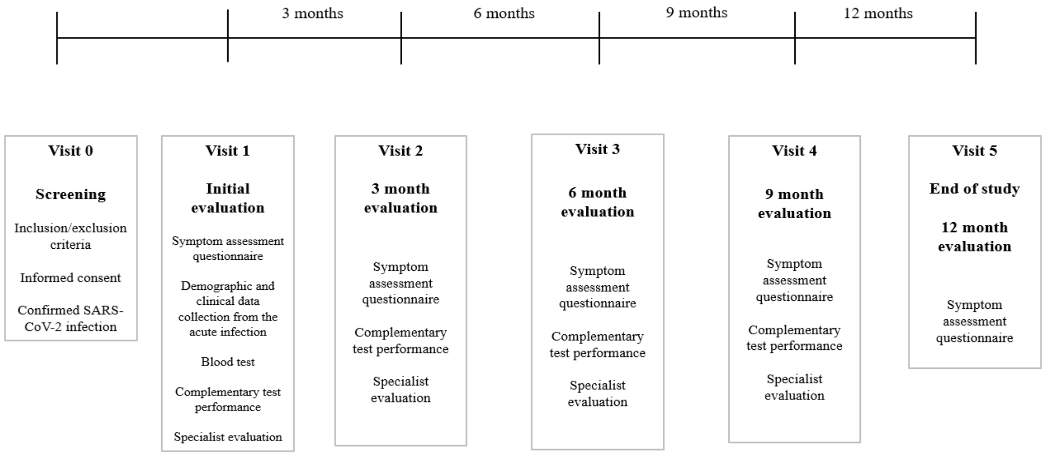

Figure S1. Follow-up protocol.

Table S1. Standardized symptom assessment questionnaire after SARS-CoV-2 infection.

**Questions**	YES	NO
Do you have temperature > 37 °C?		
Do you have anosmia or hyposmia?		
Do you have ageusia or hypogeusia?		
Do you have muscle pain?		
Do you have dyspnea?		
Do you feel fatigue?		
Do you have cough or expectoration?		
Do you have orthopnea?		
Do you feel palpitations?		
Do you have chest pain?		
Do you have hair-loss?		
Do you have any skin alterations or mouth ulcers?		
Do you have any problems walking outdoors?		
Do you have any problems walking indoors?		
Do you have any problems swallowing?		
Do you have any headaches?		
Do you feel dizzy or unstable?		
Do you have memory loss?		
Do you have audition loss?		
Do you feel anxious?		
Do you feel depressed?		
Do you feel loss of sensibility in any part of your body?		
Do you find it difficult to return to your job?		

Table S2. Outpatients laboratory results at follow-up (n=233).

**Blood test data, mean (SD)***	**Without PASC (n = 112)**	**With PASC (n = 121)**	**p value**
Creatinine (mg/dl)	0.82 (0.19)	0.79 (0.20)	0.19
Urea (mg/dl)	32.44 (11.79)	32.35 (11.28)	0.96
LDH (U/L)	182.75 (34.58)	179 (57.85)	0.67
Ferritin (ng/ml), median (p25-p75)	74 (38-151)	87 (34-169)	0.38
IL-6 (pg/ml), (p25-p75)	1.5 (1.5-3.0)	1.7 (1.5-5.0)	0.627
CRP (mg/dl)	1.01 (2.42)	0.69 (2.09)	0.34
Total proteins (g/dl)	7.01 (0.49)	7.09 (0.45)	0.25
ALT	25.69 (27.22)	24.41 (26.80)	0.75

*Data expressed as mean and standard deviation unless otherwise specified.

Abbreviations: PASC, post-acute sequelae of COVID-19; LDH, Lactate dehydrogenase; IL-6, Interleukin 6; CRP, C-reactive protein.
